# Supplementary material for: Measuring Spinal Mobility Using an Inertial Measurement Unit System: A Validation Study in Axial Spondyloarthritis
Source: Diagnostics (Basel). 2020 Jun 24;10(6):426. doi: 10.3390/diagnostics10060426 (PMC7344521; doi:10.3390/diagnostics10060426)
Supplement: Supplementary file 1 [file diagnostics-10-00426-s001.pdf]

Measuring Spinal Mobility Using an Inertial Measurement Unit System: A Validation Study in Axial Spondyloarthritis

I. Concepción Aranda-Valera [1,2,3,†], Antonio Cuesta-Vargas [4,†], Juan L. Garrido-Castro [3,5,*], Philip V. Gardiner [6], Clementina López-Medina [2,3], Pedro M. Machado [7], Joan Condell [8], James Connolly [9], Jonathan M. Williams [10], Karla Muñoz-Esquivel [8], Tom O'Dwyer [11], M. Carmen Castro-Villegas [1,2,3], Cristina González-Navas [1,2,3] and Eduardo Collantes-Estévez [1,2,3], iMaxSpA Study Group [6]

<u>SupplemenFigure S1:</u> Differences in angles measured using both systems for an individual patient.

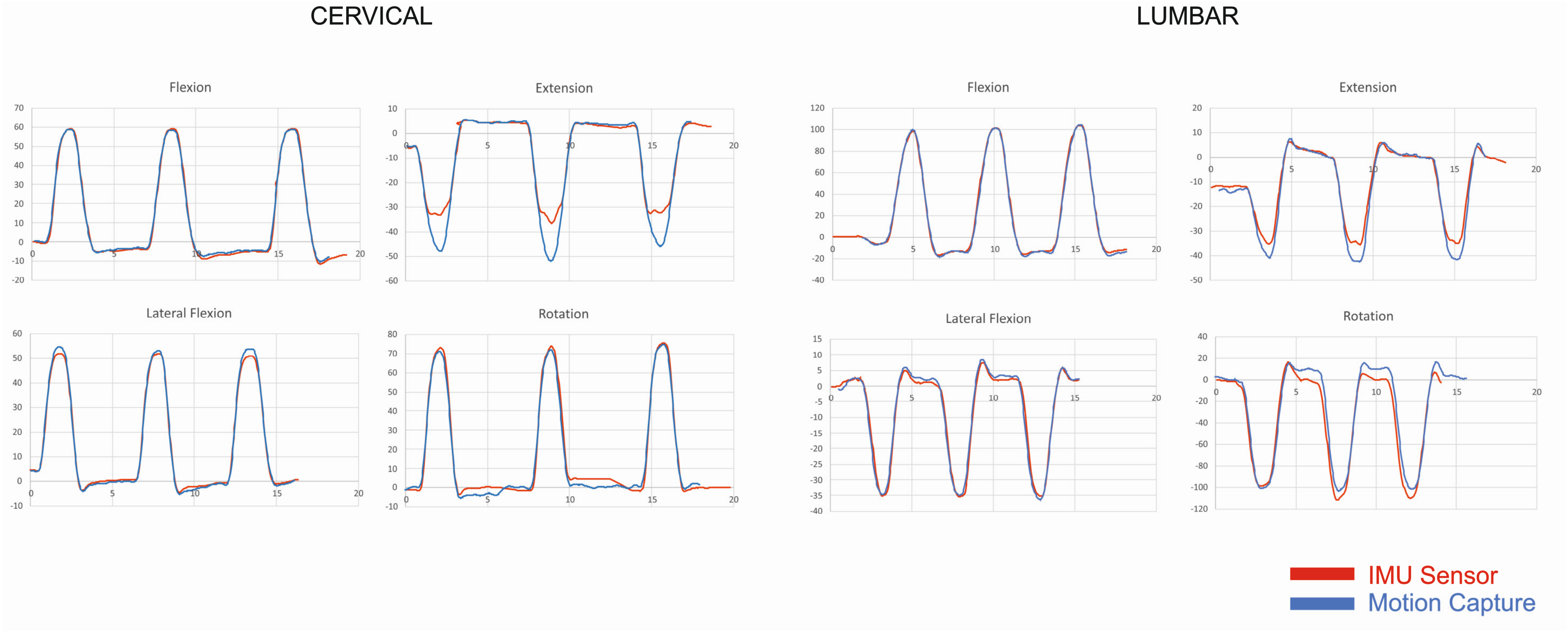

<u>Supplementary Figure S2:</u> Bland–Altman plots for cervical, L1 and lumbar region calculated analysing the differences between IMU-based system and motion capture.

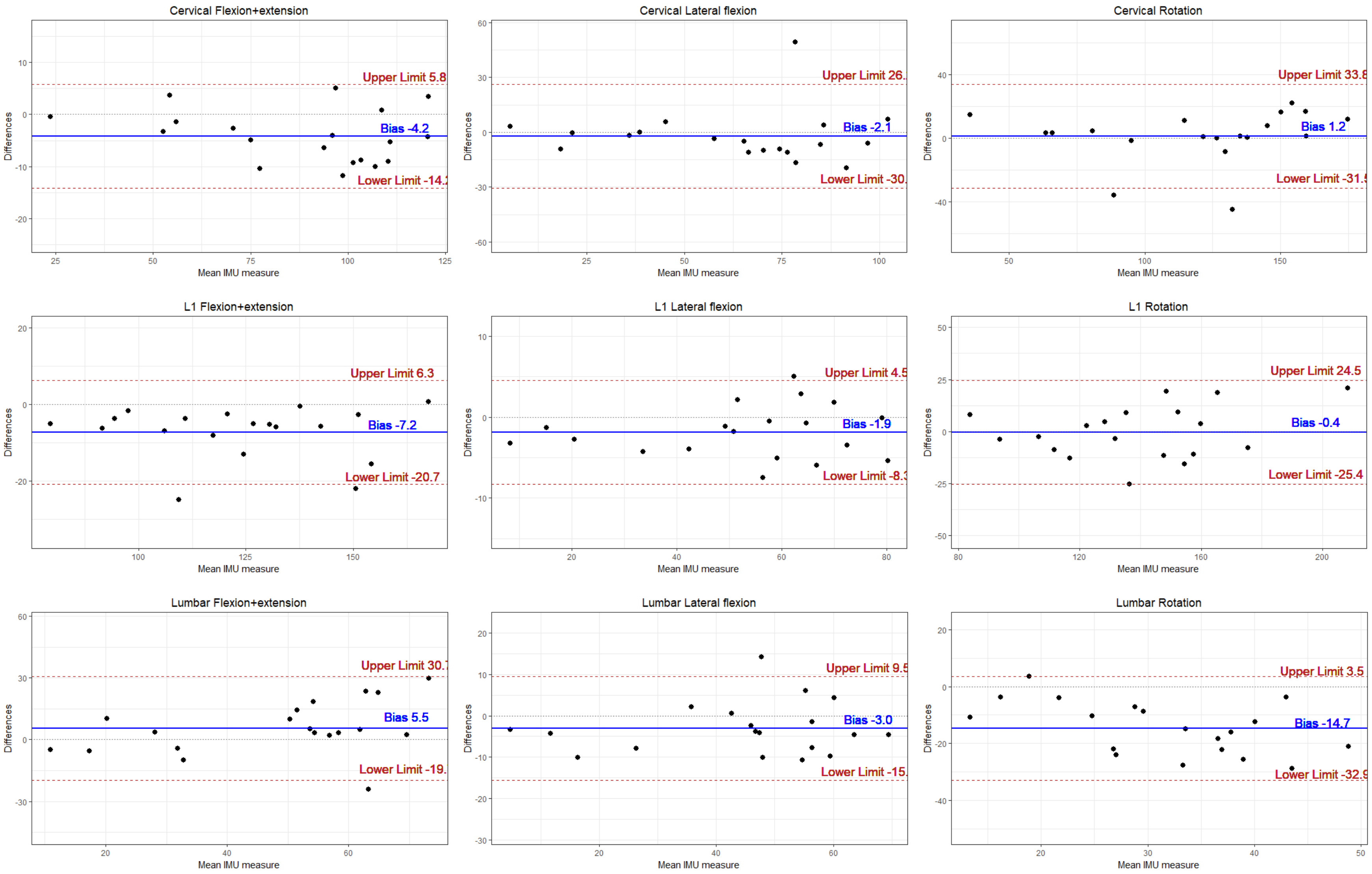

<u>Supplementary Figure S3:</u> Linear regression between the UCOASMI calculated using the motion capture and IMU-based systems.

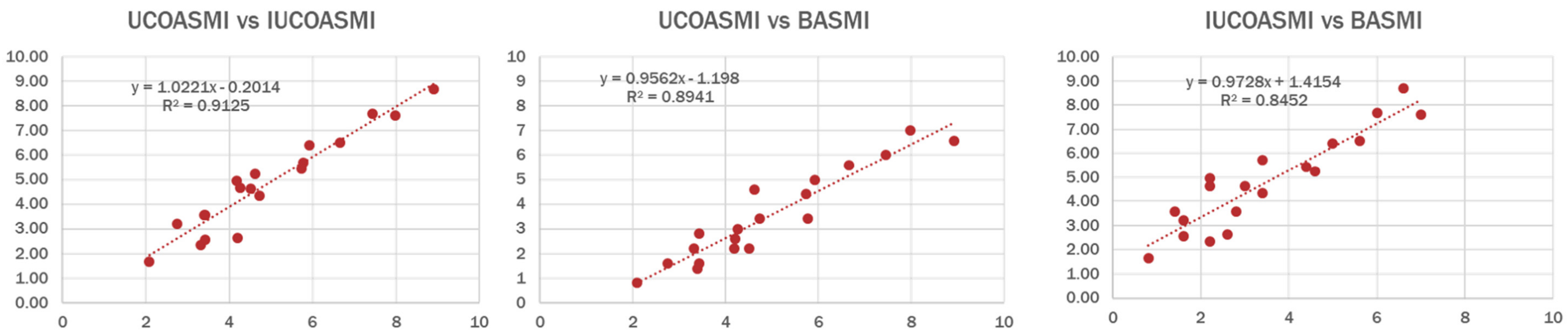

Supplementary data Figure S4: **Linear regression results comparing cervical rotation measured by a goniometer and the other automated systems.**

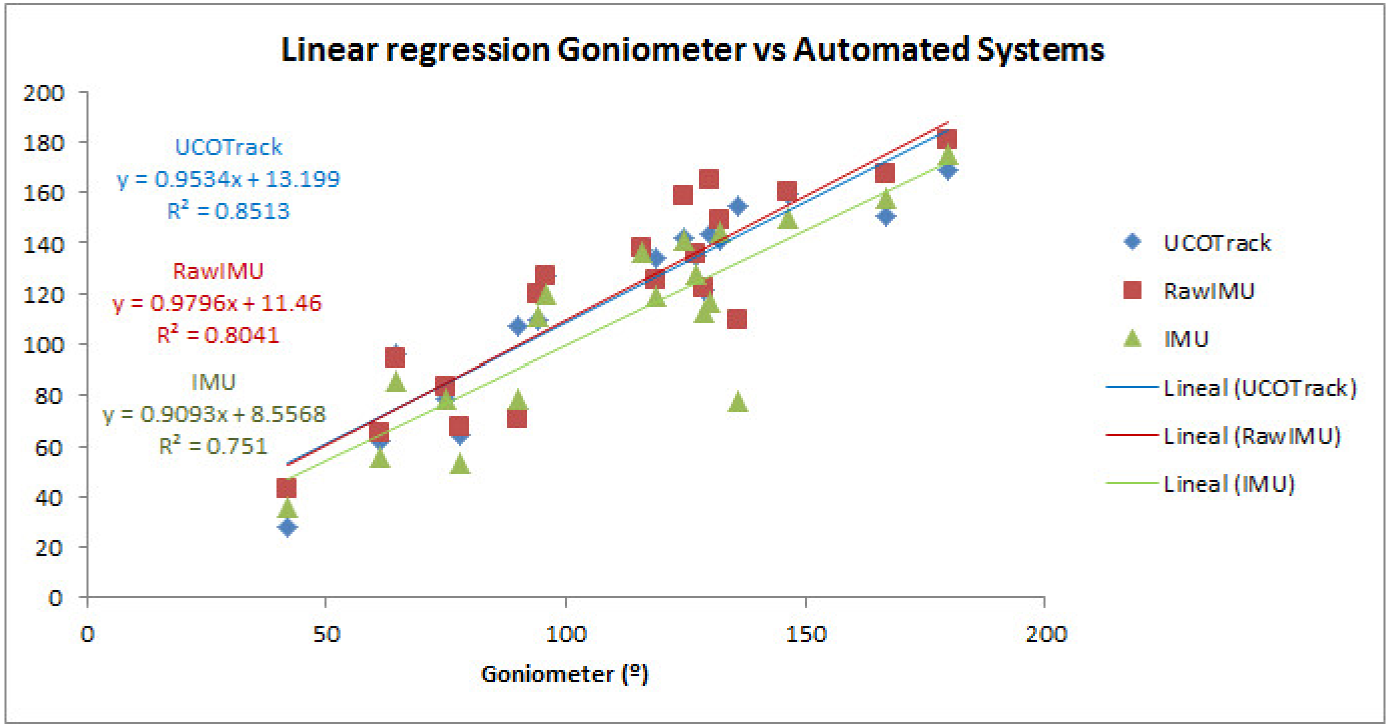

UCOTrack: results obtained by motion capture (maximum rotation of three rotation movements). IMU: results obtained by the ViMove system (mean value of three rotation movements). RawIMU: results obtained by the ViMove system (maximum rotation according to the raw results of three rotation movements).

Supplementary text T-1: Testing Protocols

1. IMU based system – ViMove

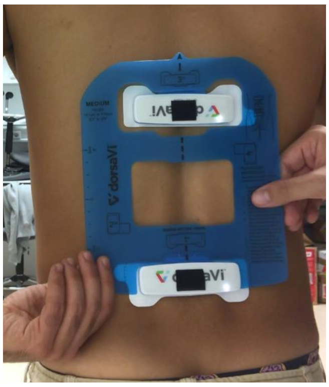

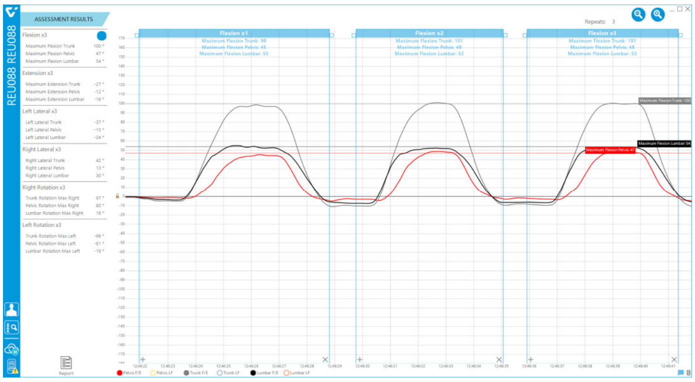

The sensors are placed on a special base provided by the manufacturer where they charge their internal batteries. In the ViMove software, the subject is added, and the type of measurement (cervical, lumbar) to be carried out is chosen. To perform the measurement, these sensors are removed from the base and placed on the patient. The sensors communicate with a recording device that is connected to the computer where the measurement is made. The movement of each sensor is represented in a graph in real-time. Each cycle of movement is identified by the system operator by pressing the 'space bar' key while the subject performs the movement. At the end of the measurement the system provides a mobility report that can be provided to the patient. Results are achieved in the internal database and the cloud.

Cervical

The upper sensor is attached to a headband that allows the sensor to be placed in the occiput (Figure 2 of the main manuscript), the lower one is placed in T3 (located by manual palpation) through an adhesive plate. The subject is asked to perform cervical movement tests which are repeated 3 times each: a maximum frontal flexion movement of the neck to return to the initial position, at a speed that is comfortable for the patient, cervical extension, rotational movements to the left and right, and lateral flexion movements, always returning to the initial position.

Lumbar

Lower sensor is placed in the sacrum, using as a reference a line drawn between the posterior superior iliac spines, above this line using specific templates depending on the height, to estimate the position of the L1 vertebra, the upper sensor is attached. Starting at standing upright in a relaxed position, the subject is asked to do a full lumbar flex motion to return to the standing position and repeat it three times. Then an extension movement, putting the arms crossed on the chest and return to the initial position, repeated three times, s performed. Similarly, the subject is asked to make lateral flexion movements to the left and right and spinal rotation to the left and right.

Results

The system provides maximum mobility ranges, for each sensor individually, and relative one to the other, for each test. There will be three cycles of each movement and the system calculates the average value of the three movement peaks. All this information is exportable in Excel © format, in addition to producing a personalized report.

More information:
https://www.dorsavi.com/wp-content/uploads/2018/12/ViMove-5.11-User-Manual-EN-INT-dV20150022k.pdf

2. Motion capture – UCOTrack

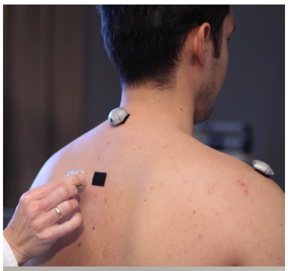

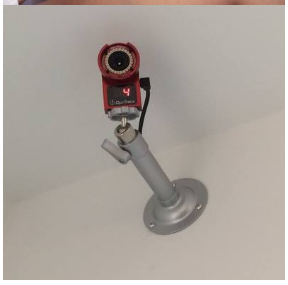

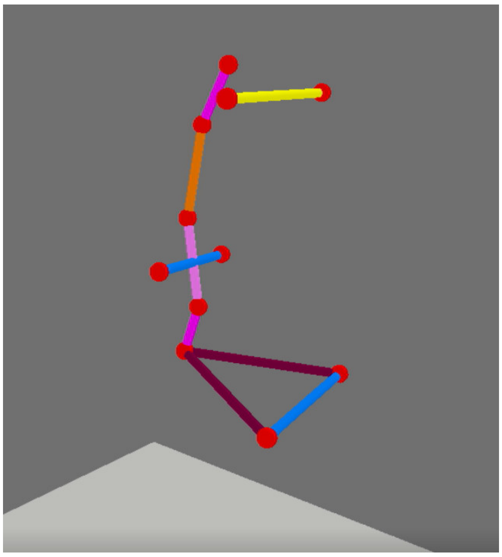

The UCOTrack motion capture system uses 4 infrared cameras and record images (50 Hz) synchronously to a central computer. Through image analysis algorithms the system recognizes 3D positions of reflective markers attached to the subject. A marker set is defined with the markers, segments, angles to measure, etc. For this study, we used a 15 reflective marker set has been defined (Figure 1 original manuscript):

1. Forehead
2. Occiput
3. Left tragus
4. Right tragus
5. clavicular acromial left
6. clavicular acromial right
7. C7
8. T12
9. S1
10. 10 cm above S1
11. midpoint between C7-S1
12. 5 cm left to L1
13. 5 cm right to L1
14. Left ASIS
15. Right ASIS

The patient must perform specific movements, such as flexion, extension, and rotation for cervical and lumbar movements. The software interprets the images and generates summary measures for marker and angles defined. For this study, the movements are the same and recorded simultaneously by both systems: the ViMove using IMU sensors and the UCOTrack using video images. The angle between the segments defined by markers 1-2 and 7-8 in the sagittal plane is analyzed to calculate cervical flexion and extension. In the same way angles of segments defined by the marker set are used for the different ROM measures. Kinematic results are filtered using a 10 Hz low pass Butterworth filter and exported to Excel© for their analysis.

<u>UCOASMI</u>

The UCOASMI index is a composite index that generates a score for cervical and lumbar mobility. It is obtained from a selection of individual measures of mobility (ROM). The score ranges from 0 to 10 (from best to worst mobility).

Measure	P=0	P entre 0 y 10	P=10
[1] Cervical frontal flexion	M1>150º	P1=10-10*(M1-20)/(150-20)	M1<20º
[2] Cervical rotation	M2>200º	P2=10-10*(M2-20)/(200-20)	M2<20º
[3] Lumbar frontal flexion	M3>200º	P3=10-10*(M3-60)/(200-60)	M3<60º
[4] Lumbar lateral flexión	M4>120º	P4=10-10*(M4-20)/(120-20)	M4<20º
[5] Lumbar rotation	M5>160º	P5=10-10*(M5-0)/(160-0)	M5<0º

UCOASMI = (P1 +P2 +P3 +P4 +P5)/10

More information:

http://www.ucotrack.es

Assessment of spinal mobility in ankylosing spondylitis using a video-based motion capture system. Man Ther. 2012;422–6.

Validation of a new objective index to measure spinal mobility: The University of Cordoba Ankylosing Spondylitis Metrology Index (UCOASMI). Rheumatol Int. 2014;401–6.
